# A case of subclinical Cushing's syndrome in pregnancy with superimposed preeclampsia

**DOI:** 10.1002/ccr3.2592

**Published:** 2020-02-03

**Authors:** Eriko Eto, Takashi Mitsui, Shoko Tamada, Jota Maki, Kei Hayata, Hisashi Masuyama

**Affiliations:** ^1^ Department of Obstetrics and Gynecology Dentistry and Pharmaceutical Sciences Okayama University Graduate School of Medicine Okayama Japan

**Keywords:** adrenal incidental tumor, pregnancy, subclinical Cushing's syndrome, superimposed preeclampsia

## Abstract

When we see preexistent hypertension in pregnancy, subclinical Cushing's syndrome should be considered in the differential diagnosis since this disorder can cause perinatal complications. MRI can be useful for identifying adrenal incidental tumors during pregnancy.

## Graphical abstract

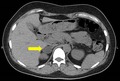

## 1 CASE REPORT

Patients with adrenal adenomas can develop subclinical Cushing's syndrome (SCS) due to an excess of cortisol production, without the typical clinical signs and symptoms of Cushing's syndrome (CS).^1^ SCS often co‐occurs with some diseases like obesity, diabetes mellitus, and hypertension.^2^ When a woman's pregnancy is complicated by SCS, the disease course is not well known as SCS during pregnancy is rare. Here, we report a case of a pregnant woman diagnosed with SCS during the pregnancy, complicated with superimposed preeclampsia.

**Figure 1:**
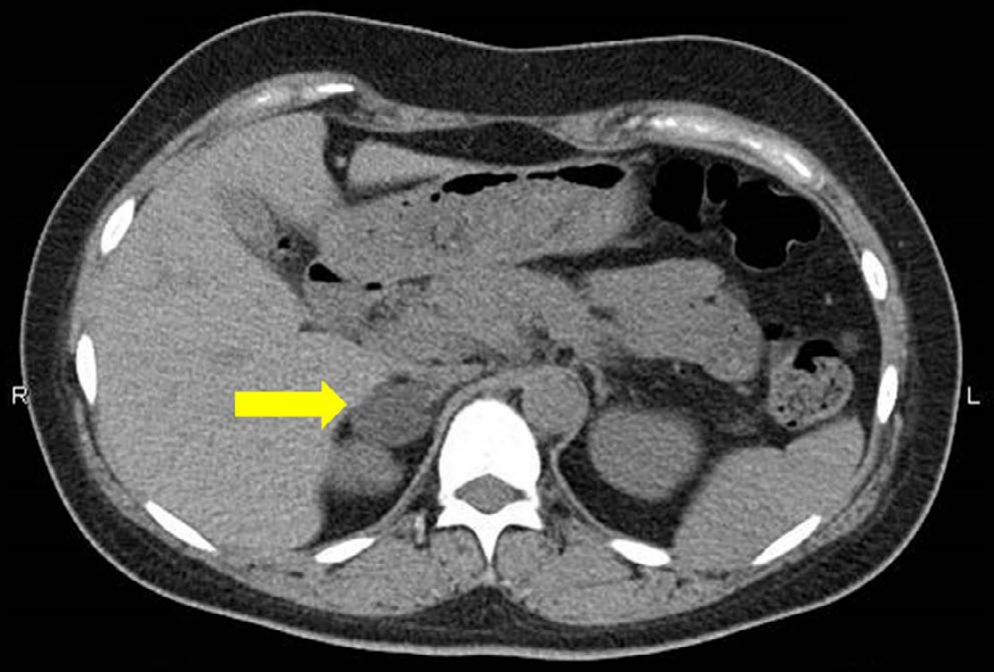
Computer tomography on postpartum day 3 (The yellow arrow shows a 28 mm × 28 mm right adrenal mass.)

**Figure 2:**
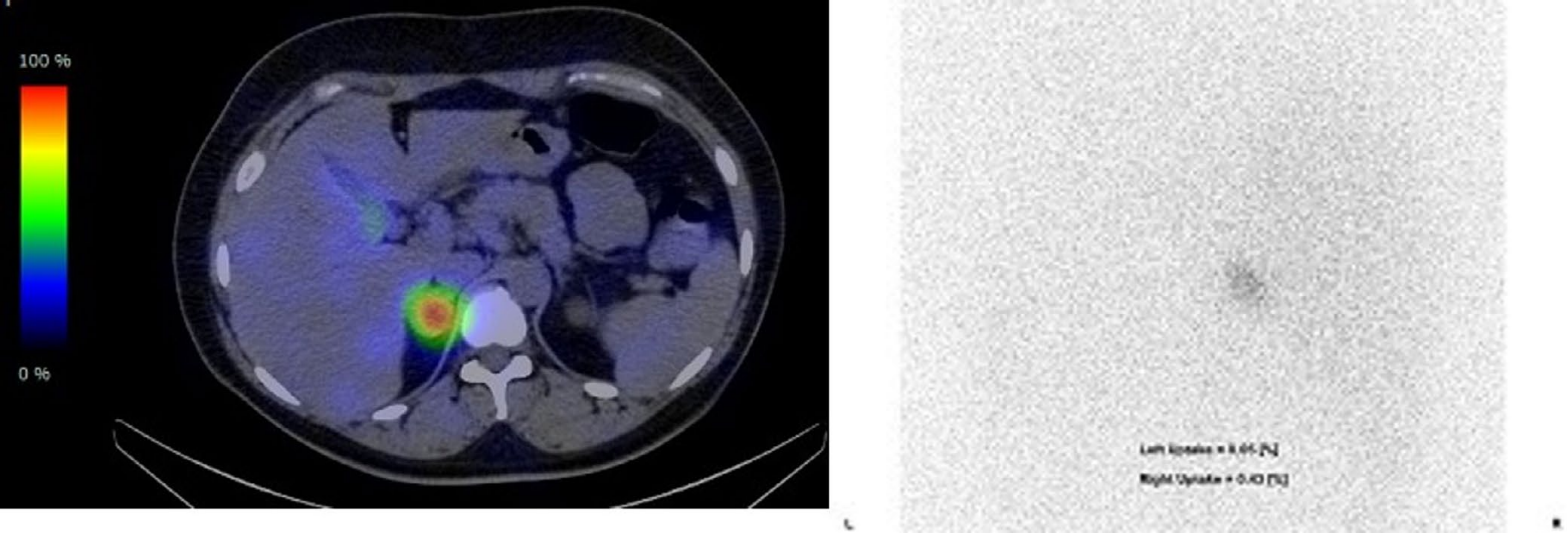
Adosterol adrenal scintigraphy on 2 months postpartum (There is an uptake only on the tumor side.)

A 34‐year‐old woman, 3 gravida 2 para, was referred to our institution at 21 weeks of gestation with hypertension. She had no previous medical history. She had a normal blood pressure during her past two pregnancy. However, for her current pregnancy, her blood pressure suddenly raised to 170/91 mm Hg at 12 weeks of gestation. Physical examination at hospitalization revealed a blood pressure of 170/110 mm Hg and a body mass index of 31.6 kg/m^2^. No clinical signs like buffalo hump, moon face, and striae were observed. Following hospitalization, her blood pressure ranged between 130‐140 and 90‐100 mm Hg with antihypertensive therapy (Nifedipine 40 mg/day). The 24‐hour urine total protein level was checked few times and showed normal data (198.0‐293.2 mg). We suspected preexisting hypertension. Hormonal profiles revealed a normal serum cortisol level of 15.5 (normal range 4.5‐21.1 μg/dL) and a low plasma level of adrenocorticotropic hormone (ACTH) early in the morning of ＜1.5 (normal range 7.2‐63.3 pg/mL). SCS was suspected. Following this, the total 24‐hour urine protein level was found to be 11 g/day at 26 weeks of gestation, and diagnosis of superimposed preeclampsia was made. Blood test revealed low platelets (11.6 × 10^4^/μL), elevated lactate dehydrogenase (437 U/L), and low AT3 (75%) at 27 weeks of gestation. The estimated fetal weight by ultrasonography was 820 g (−1.21 SD) at 25 weeks, 868 g (−0.97 SD) at 26 weeks, and 846 g (−1.82 SD) at 27 weeks of gestation. The newborn was delivered at 28 weeks of gestation because of maternal partial hemolysis, liver enzymes, and low platelet count occurring in pregnancy (HELLP) syndrome and fetal growth arrest. A female infant weighing 793 g with Apgar score of 6 at 1 minute and 8 at 5 minutes was delivered by cesarean section. On postpartum day 3, computer tomography was performed and revealed a 28 mm × 28 mm right adrenal mass (Figure 1). Two weeks postpartum, hormonal profiles showed a normal serum cortisol level (6.1 μg/dL) and a low plasma level of ACTH early in the morning (2.6 pg/mL) again. Serum cortisol after an overnight 1 mg dexamethasone suppression test was 3.3 μg/dL, and the ACTH level early in the morning was low (＜1.5 pg/mL), 6 weeks following the delivery. We confirmed the diagnosis of SCS. Her blood pressure remained at 130‐140/80‐90 mm Hg, her creatinine level was 0.6‐0.7 mg/dL, and urine protein/creatinine ratio was 0.7‐0.9 after the delivery. Adosterol adrenal scintigraphy was performed 2 months postpartum (Figure 2). Uptake was observed only on the tumor side. A laparoscopic adrenalectomy is proposed to the patient. No neurodevelopmental difficulties have been identified in the infant.

There are serious maternal and fetal complications during pregnancy with CS, such as hypertension, gestational diabetes, heart failure, spontaneous abortion, preterm labor, and fetal growth retardation.^3^ However, it is unclear whether such complications occur in pregnancies with SCS. The diagnosis of SCS during pregnancy is challenging, as in the case of CS, due to the similarities of some of its features with that of a normal pregnancy, accompanied by physiological hypercortisolism.^4^ Estrogen increase can promote cortisol binding coupled with increases in placental ACTH and corticotrophin‐releasing hormone can lead to increases in free cortisol levels in plasma and the uterus.^3^ Moreover, SCS is asymptomatic, and the activity of the tumor varies among patients.^1^ This case was complicated by hypertensive disorder of pregnancy in the first trimester; however, the patient showed no sign of preeclampsia at this time. Following this, we suspected preexistent hypertension. The differential diagnosis for the cause of hypertension was an adrenal tumor, renovascular abnormalities and thyroid disease, and so on. We investigated her adrenal function, thyroid function, and renal function by blood tests not only during pregnancy but also 2 and 6 weeks postpartum. Abnormal adrenal morphology and renovascular abnormalities were not detected by ultrasonography during pregnancy. To identify the adrenal incidental tumor, MRI may be a better alternative than ultrasonography during pregnancy.

Laparoscopic adrenalectomy has become a standard approach for adrenal tumors.^1^ There are some studies that recommend surgical treatment for the management of CS during pregnancy.^3^, ^5^ A surgical approach could be effective for SCS as well. In this case, the condition of SCS could have improved if the adrenal tumor was found and laparoscopic adrenalectomy was performed during the pregnancy.

In conclusion, serious perinatal complications can occur due to SCS in pregnancy, such as presented in this case of CS. There is no consensus on the management of SCS during pregnancy due to the rarity of this condition. We hope this report will contribute to the evidence for understanding SCS.

## CONFLICT OF INTEREST

The authors have no conflict of interest to declare.

## AUTHOR CONTRIBUTIONS

EE: designed the study and wrote the initial draft of the manuscript. TM: contributed to analysis and interpretation of data, and assisted in the preparation of the manuscript. All other authors: have contributed to data collection and interpretation, and critically reviewed the manuscript. All authors: approved the final version of the manuscript and agree to be accountable for all aspects of the work in ensuring that questions related to the accuracy or integrity of any part of the work are appropriately investigated and resolved.
